# A study protocol for quantifying patient preferences in neuromuscular disorders: a case study of the IMI PREFER Project

**DOI:** 10.12688/wellcomeopenres.16116.1

**Published:** 2020-10-23

**Authors:** Aura Cecilia Jimenez-Moreno, Cathy Anne Pinto, Bennett Levitan, Chiara Whichello, Christine Dyer, Eline Van Overbeeke, Esther de Bekker-Grob, Ian Smith, Isabelle Huys, Jennifer Viberg Johansson, Kate Adcock, Kristin Bullock, Vikas Soekhai, Zhong Yuan, Hanns Lochmuller, Ardine de Wit, Grainne S. Gorman

**Affiliations:** 1 Wellcome Centre for Mitochondrial Research, Newcastle University, Newcastle-Upon-Tyne, NE2 4HH, UK; 2 Patient Centered Research, Evidera, London, W6 8BJ, UK; 3 Pharmacoepidemiology Department, Centre for Observational and Realworld Evidence, Merck & Co, Inc., Rahway, NJ, USA; 4 Department of Epidemiology, Janssen Research & Development, Titusville, NJ, USA; 5 Erasmus School of Health Policy & Management and Erasmus Choice Modelling Centre, Erasmus University Rotterdam, Rotterdam, The Netherlands; 6 Department of Clinical Pharmacology and Pharmacotherapy, University of Leuven, Leuven, Belgium; 7 Julius Center for Health Sciences and Primary Care, University Medical Center Utrecht, Utrecht, The Netherlands; 8 Centre for Research Ethics & Bioethics, Uppsala universitet, Uppsala, 75122, Sweden; 9 Muscular Dystrophy UK, London, UK; 10 Global Patient Safety Department, Eli Lilly & Co., Indianapolis, IN, 46205, USA; 11 Brain and Mind Research Institute, University of Ottawa, Ottawa, Canada

**Keywords:** Myotonic Dystrophy, mitochondrial disease, risk tolerance, Best Worst Scaling, Discrete Choice Experiment, Q-methodology, patient preferences, IMI-PREFER, treatment preferences.

## Abstract

**Objectives:** Patient preference studies are increasingly used to inform decision-making during the medical product lifecycle but are rarely used to inform early stages of drug development.  The primary aim of this study is to quantify treatment preferences of patients with neuromuscular disorders, which represent serious and debilitating conditions with limited or no treatment options available.

**Methods:** This quantitative patient preferences study was designed as an online survey, with a cross-over design.  This study will target two different diseases from the neuromuscular disorders disease group, myotonic dystrophy type 1 (DM1) and mitochondrial myopathies (MM). Despite having different physio-pathological pathways both DM1 and MM manifest in a clinically similar manner and may benefit from similar treatment options.  The sample will be stratified into three subgroups: two patient groups differentiated by age of symptom onset and one caregivers group.   Each subgroup will be randomly assigned to complete two of three different preference elicitation methods at two different time points: Q-methodology survey, discrete choice experiment, and best-worst scaling type 2, allowing cross-comparisons of the results across each study time within participants and within elicitation methods. Additional variables such as sociodemographic, clinical and health literacy will be collected to enable analysis of potential heterogeneity.

**Ethics and Dissemination:** This study protocol has undergone ethical review and approval by the Newcastle University R&D Ethics Committee (Ref: 15169/2018). All participants will be invited to give electronic informed consent to take part in the study prior accessing the online survey. All electronic data will be anonymised prior analysis. This study is part of the Patient Preferences in Benefit-Risk Assessments during the Drug Life Cycle (IMI-PREFER) project, a public-private collaborative research project aiming to develop expert and evidence-based recommendations on how and when patient preferences can be assessed and used to inform medical product decision making.

## Study highlights

- Neuromuscular disorders represent uncommon conditions with limited treatment options and patient preferences studies may inform early medical product development decisions
- This study aims to quantify patient treatment preferences including variables such as relative importance and benefit to risk trade-offs
- This study will compare feasibility, level of understanding and validity of results between three different preference elicitation methods
- This study aims to be a large patient preference study targeting rare disease groups through an international collaboration across different patient organizations

## Introduction

Neuromuscular diseases (NMD) represent uncommon ^1^, serious and debilitating (i.e. muscle weakness) conditions; all progressive with poor prognosis and with limited or no treatment options available. For this case study, we have selected two neuromuscular disorders (i.e. myotonic dystrophy type 1 [DM1] and mitochondrial myopathies [MM]) that despite having different physio-pathological pathways both manifest in a clinically similar manner. DM1 and MM together affect around 20 people in every 100,000 worldwide ^1, 2^. DM1 is the most prevalent type of NMD in adults and is the result of an autosomal genetic defect ^3^. MM are the result of either inherited or spontaneous mutations in either the mitochondrial genetic material or the nuclear genetic material that result in affected cellular mitochondria ^4^.

In both diseases, different body tissues and organs may be affected in function or during development resulting in a multisystem and heterogeneous phenotype. DM1 and MM can affect more than one family member or generation due to their pattern of inheritance, and onset of symptoms can occur at different stages of life (i.e. from early childhood to late adulthood). In addition to muscle weakness, both DM1 and MM commonly affect the central nervous system (CNS), with symptoms such as: impaired or reduced cognitive function (i.e. attention, visual-spatial function, logical memory, processing speed) learning difficulties, daytime sleepiness and fatigue, and mobility limitations ^5– 7^. General cognitive deficits have been described in over 60 to 70% of patients and the prevalence and severity depends on the age at onset of the disease, with patients with symptoms established earlier in life more prone to this phenotype ^5– 8^. People affected by NMD diseases may have limited functional capacity to perform certain daily activities and often require caregiver assistance. This adds to the complexity of studying the NMD population given that patients may rely significantly on caregivers for relevant care-related decisions ^9, 10^.

There is no specific cure for either disease, and standard of care offers few options for managing symptoms, although potential new treatments targeting these diseases are emerging or in phase I and phase II of the drug development process ^4^. There are a number of challenges in developing treatment in the context of rare diseases such as NMD patient preference information (PPI) can better inform decision-makers (e.g. industry, regulators and reimbursement agencies) regarding the unmet needs of the patient population and potential value of new treatment developments, relevant clinical trial endpoints, and benefit-risk decision-making processes across the medical product lifecycle. PPI is also especially important, as clinical trial evidence is often considerably uncertain or variable given the rare nature of the disease and challenges affecting trial enrolment ^11– 13^. Although recent research has been conducted to better understand patient preferences for NMD treatments (pharmacological and non-pharmacological) ^11, 14– 18^, it has consisted mainly of qualitative research and little has been done to objectively quantify preferences for pharmacologic interventions ^19^.

Given the important role of caregivers in this disease area, they also play a central role in this study and allow us to capture important priorities and treatment attributes for patients, which may otherwise go unrecognized, and compare preferences obtained directly by patients with preferences as elicited from caregivers, on behalf of the patient they care for.

## Study protocol

This study is part of the Patient Preferences in Benefit-Risk Assessments during the Drug Life Cycle (IMI-PREFER) project, a public-private collaborative research project aiming to develop expert and evidence-based recommendations on how and when patient preferences can be assessed and used to inform medical product decision making. In particular, this study was designed to answer two types of research questions: clinical and methodological. The clinical questions were developed by a team of experts in healthcare and research of NMD and was inspired by advice from the patient representatives. The methodological questions were selected from those generated as part of the PREFER project initial tasks.

### Specific objectives

1. Primary aims

- Clinical: To elicit and quantify patient preferences, including benefit to risk trade-offs (e.g. relative importance, minimum acceptable benefit (MAB), maximum acceptable risk (MAR)) for future NMD treatments.
- Methodological: To describe and compare results obtained from three different preference assessment methods; to conduct intra-methods comparability to analyse relatively simpler versus more complex preference elicitation methods.

2. Secondary aims

- Clinical:
  - To describe and compare preferences among the different participant subgroups (i.e. disease type, disease phenotypes, patients and caregivers):
    ◦ To assess how generalizable preferences (i.e. relative importance, MAB and MAR) are from one specific disease to a different disease but with similar clinical characteristics (i.e. DM1 and MD);
    ◦ To understand the degree to which patients’ preferences and caregiver preferences align with each other;
    ◦ To identify clinically meaningful subgroups based on the association between specific preferences and specific demographic and clinical characteristics.
  - To describe heterogeneity of responses resulting from different preference elicitation methods for patients and caregivers based on demographics and medical history (e.g. patients disease severity; prior treatment history; and, genetic status of caregivers).
  - To demonstrate the feasibility and acceptability of assessing PPI in a population that may have varying levels of cognitive limitations.
- Methodological:
  - To describe and compare responses (e.g. preference results, heterogeneity in responses, compliance and level of understanding) from three different preference elicitation methods applied within the same disease population.
  - To describe and compare responses reliability and preference variability between two related diseases with similar clinical characteristics (i.e. DM1 and MD patients).
  - To describe and compare preferences of two different types of stakeholder groups (i.e. comparing patient preferences with caregivers’ judgments regarding patient preferences).
  - To describe associations between responses from a preference elicitation method and results obtained from psychosocial constructs assessments.

### Study design

**Figure 1.**
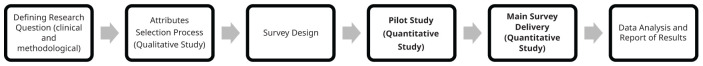
Case Study Research Approach.

Figure 1 presents the overall approach of this research and highlights the research stage where this protocol sits.

Prior this study, a separate qualitative study was conducted to understand what was important to patients and to help define attributes and attribute levels that form part of this protocol’s survey. The qualitative study included 52 participants (patients and caregivers) who completed in-person semi-structured interviews and focus group discussions using an interview guide. The findings from this study have been described before.

The qualitative study findings became the main source of data to inform this study’s attribute and attribute levels. Additional evidence collected during the literature review ^11, 14– 18^, and experience-based opinions from the key members of the team (patients, clinical experts and methodological experts) were also considered when designing the survey instruments.

Soekhai *et al*. ^20^ identified 32 different preference methods and divide them into two main groups, exploration (applicable for qualitative studies) and elicitation (applicable for quantitative studies). For our qualitative study we included semi-structured individual interviews and focus groups. For this study we chose three different elicitation methods, Best-worst Scaling type 2 (BWS2) and Q-methodology from the ranking methods subgroup and discrete-choice experiment (DCE) from the discrete-choice-based methods. These three methods are described in detailed later in this protocol. The reason for choosing three different methods has been to explore alternatives to elicit patient preferences in rare diseases at early stages of the drug development lifecycle where little is known about potential treatment attributes and where cognitive limitations and cognitive fatigue may be present.

Prior to the final quantitative survey, presented here, pilot testing was performed to ensure comprehension and acceptability of the surveys and other study materials. Results of the pilot testing were used to finalize the research materials for the main survey and to obtain preliminary preference data to further inform the experimental design and sample size calculation for the DCE tool as core method of our study.

**Figure 2.**
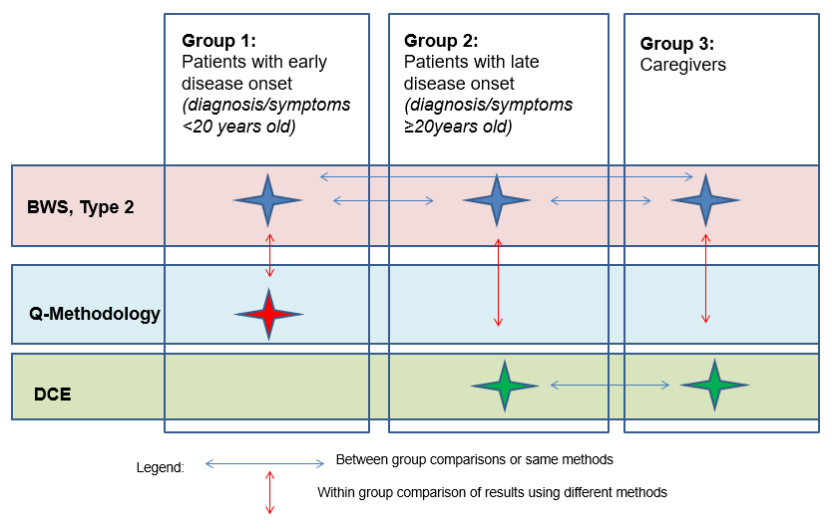
Quantitative study design and patient preference elicitation methods allocation between sample groups. BWS: best-worst scaling; DCE: discrete-choice experiment.

This quantitative study will include both patients and caregivers. Participants will be asked to complete an online survey that will be administrated at two different time points (T1 and T2) with a 2-week period in between. Only the first online survey (T1) will include demographic, clinical history questions and a patient reported outcome as surrogate of disease severity. Then, each time point (T1 and T2) will include: psychosocial construct measures (health literacy and numeracy), and one preference elicitation method (or exercise). The preference elicitation exercise to complete will be based on the group each participant gets allocated to. If allocated to Group 1, the survey at each time point will include one of the two methods designated as being simpler (i.e. BWS2 or Q-methodology), while those allocated to Group 2 will be given one of the more complex methods instead of the Q-methodology (i.e. DCE) ( Figure 2).

Figure 2 presents the cross-over design planned for this study. This design will also allow patients and caregivers to act as their own control allowing comparison of results from one assessment time point (T1) to the other time point (T2), thereby reducing the cognitive burden associated with testing the two methods of interest at the same time point. The washout period between T1 and T2 (i.e. 2 weeks) will reduce the potential bias of a learning effect and no major changes in disease status are expected in such a short period of time.

This study was supported by four patient representatives who actively participate in study team meetings and in ad hoc decisions needed throughout the study design process ^19^.

Online cross-over survey using two different preference elicitation methods:

- Group 1 (DM1 and MM patients with early disease onset; i.e. self-reporting initial symptoms before turning 20 years old)
  - BWS2 and Q-methodology
- Group 2 (DM1 and MM patients with late disease onset; i.e. self-reporting initial symptoms at 20 years old or older
  - BWS2 and DCE
- Group 3 (Caregivers of patients with DM1 and MM)
  - BWS2 and DCE

***Comparisons.*** Within group and between group comparison of preferences obtained using different preference elicitation methods will be evaluated, as illustrated in Figure 1 below:

### Participant identification, recruitment and follow-up

Participants will be recruited via patient organisations and patient registries in the UK and four other countries. The list of recruiting partners follows: Muscular Dystrophy UK (MDUK) Myotonic Dystrophy Support Group (MDSG) Mito Foundation; Cure DM CIC; the Lily Foundation for mitochondrial disorders (Lily); United Mitochondrial Disease Foundation (UMDF); Muscular Dystrophy Canada (MDC); Muscular Dystrophy Association (MDA); Myotonic org; MitoCanada; Muscular Dystrophy New Zealand (MDNZ); and, via the UK Myotonic Dystrophy Patient Registry and the New Zealand Neuromuscular Disease Patient Registry (see *Extended data* for materials given to participants ^21^).

### Inclusion criteria

All participants must be 18 years of age or older and have an active email account to register for the online survey. Additional criteria for patients and caregivers are listed below.

***Patients***

- Group 1: self-reported as diagnosed with DM1 or MD patients with early onset of disease (established diagnosis or first reported symptoms before 20 years of age).
- Group 2: self-reported as diagnosed with DM1 or MD patients with late onset (established diagnosis or first reported symptoms on or after 20 years of age)

***Caregivers***

- Group 3: caregiver defined as a spouse, partner, parent, legal guardian, close relative, or other adult close to the family living either in the same house or in contact with the DM1 or MD patient in a caregiver relationship at least 4 times/week for at least one hour or more per day.
- Caregivers can be carriers of the genetic mutation or non-carriers (or diagnosed with the disease themselves), but at the time of interviewing they would report being a caregiver.
- Caregivers will be asked to contribute based on their judgments regarding the needs of the patient they care for and not about their own personal preferences. Caregivers will allow representation of patients that otherwise could not participate themselves (e.g. pediatrics, cognitively affected, deceased).

### Exclusion criteria

- Participants recognising unable to provide informed consent or to complete online questionnaires.
- Participants reporting historical diagnoses of encephalopathy or dementia (as these may indiscriminately have a significant impact on cognitive skills and ability to complete the survey regardless the age of initial symptoms)

### Ethics and dissemination

As part of promotion and recruitment process, invitation letters will be distributed via the Patient Organizations partnered with this study. Those interested in participating will be invited to complete a short questionnaire to verify their interest in participating (i.e. electronic consent form) and to determine whether they meet the inclusion criteria based on their demographic characteristics and self-reported established diagnosis. A participant information sheet (PIS) with the study and data handling details will be sent to potential candidates as part of the initial invitation letter. The study will be conducted according to the Guidelines for Good Clinical Practice (GCP) throughout the study. Accidental or unexpected deviations from the protocol (as approved by the Newcastle Ethics committee; ID: BH162126) will be properly followed. The study will be conducted in accordance to the UK Policy Framework for Health and Social Care Research and the new EU General Data Protection Regulation (GDPR). Informed consent materials and PIS are available as *Extended data* ^21^.

### Survey measures

***Demographics questionnaire. *** A brief set of demographical data will be collected from participants, including details such as: age, gender, relationship to the patient (only applicable for caregivers), educational degree and work status.

***Patient reported outcome (PRO) to assess participation in daily life activities. *** The ACTIVLIM questionnaire is a Rasch model assessment tool that measures limitations in the execution of daily life activities that a patient might face whatever the strategies involved. The ACTIVLIM questionnaire has been calibrated for use in children and adults (from 6 to 80 years old) with neuromuscular disorders in which respondents score the level of experienced difficulty when performing daily activities as judged by the parents of the affected children or by the adult patients themselves ^22, 23^. This scale includes 22 daily activities (specific activities for children, 4 specific activities for adults and 14 common activities) and each item is scored on a 3-level scale (impossible, difficult, easy).

***Health literacy and numeracy.*** Psychological constructs will inform the results by providing information about the patients that can affect their individual health-care (e.g. treatment) decisions ^24^. The assessments selected for this study are:

1) Chew’s Set of Brief Screening Questions ^25^

This three-question tool assesses perceived health literacy and confidence in reading, filling and learning from health care related written materials. This type of questionnaire assesses the degree to which individuals have the capacity to obtain, process and understand basic health information in order to make appropriate health decisions.

2) Subjective Numeracy Scale ^26^

This measures the perceived ability to perform three different numerical tasks and is scored with a 6-point Likert-type scale: (1). How good are you at working with fractions? (“Not good at all, 1” to “Extremely Good, 6”); (2) How good are you at figuring out how much a shirt will cost if it is 25% off? (“Not good at all, 1” to “Extremely Good, 6”); and (3) How often do you find numerical information to be useful? (“Never, 1” to “Very Often, 6”). The first two questions focus on self-reported numeracy skills, while the third focuses on subject preference.

**Figure 3.**
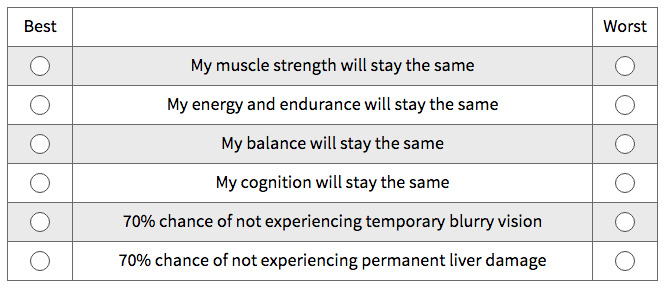
Best Worst Scaling Type 2 or Case 2 Best Worst Scale - choice task example.

***Patient preference elicitation methods.*** As mentioned above, this study has been designed as a cross-over study to allow within and between group comparison of the patient preference elicitation methods (or exercises) to be evaluated as part of the methodological objectives as illustrated in Figure 3. As explained before, the selection of these methods originates from research performed as part of the PREFER project ^20^. An example of each elicitation method is available as Extended data ^21^. A brief explanation of the patient preference elicitation methods is provided below.

- Patient preference elicitation method 1: BWS2

BWS2 is a method on which respondents are asked to consider a treatment profile which is described by multiple attributes with specific levels (e.g. 25% risk of mild side effects or 1% risk of severe side effects). Based on the presented attribute levels respondents select their best and worst attribute level. This method has become more and more popular in health preference research since it can uncover attribute level importance and ranking, reduce cognitive burden of the elicitation task by focusing on one profile at a time and is relatively easy to design ^27, 28^. Figure 3 shows an example case 2 BWS choice task with six attributes each with a specific level from which respondents make their best and worst choices.

- Patient preference elicitation method 2: Q-methodology

**Figure 4.**
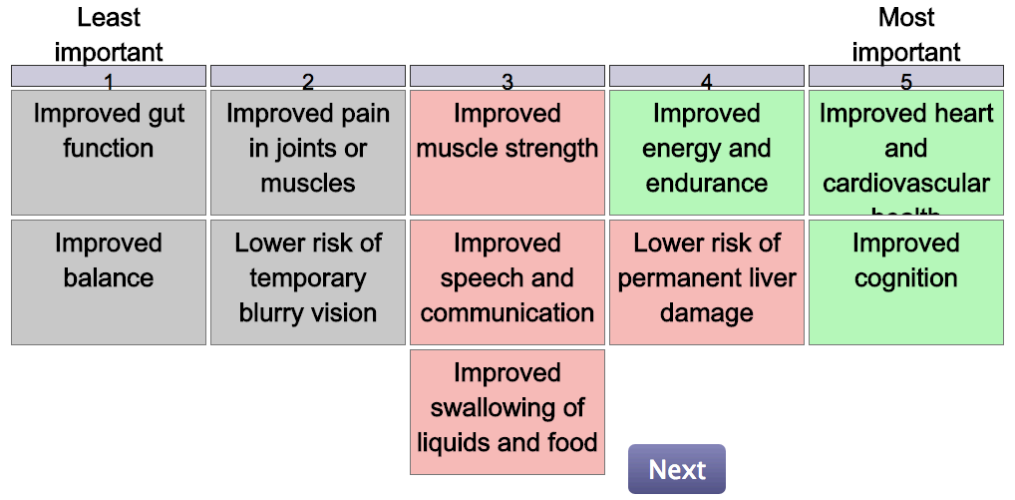
Q-methodology – sorting grid example.

This method asks participants to rank statements (in this case referring to treatment ‘attributes’) on a special V-shaped grid, according to whether, or how strongly, they feel the statement is important to them ( Figure 4). The list of statements is called the Q-set and describes a variety of treatment attributes. Through an online survey, participants will be presented with each statement one-by-one, asked to read it, and then place it into one of three different piles: ‘Most important, ’I’m not sure’ as Neutral, or ‘Least important’ representing how strongly they feel the attribute is important to them. This serves as a step through which the participants are able to familiarise themselves with the attributes and gives them a chance to make initial assessments. After sorting all the Q-set into three piles, they are then asked to rank the statements in the -V-shaped grid. Next, they are asked to rank the statements they categorised as ‘Most Important’ at the right end of the grid. The statements they felt were ‘Least Important’ are ranked at the left of the grid, with the ‘I’m not sure’ statements in the middle, indicating neutrality. Through these individual rankings, an individual’s unique viewpoints or subjective experiences can be captured, which is called a Q-sort. The Q-sorts of a group of participants are then statistically correlated in order to reveal clusters of similar viewpoints ^29^. Additionally, at the end of the exercise, in an open-question format, participants will be queried to explain the rationale behind the attributes selected as ‘most important’ and the ones selected as ‘least important’.

- Patient preference elicitation method 3: DCE

**Figure 5.**
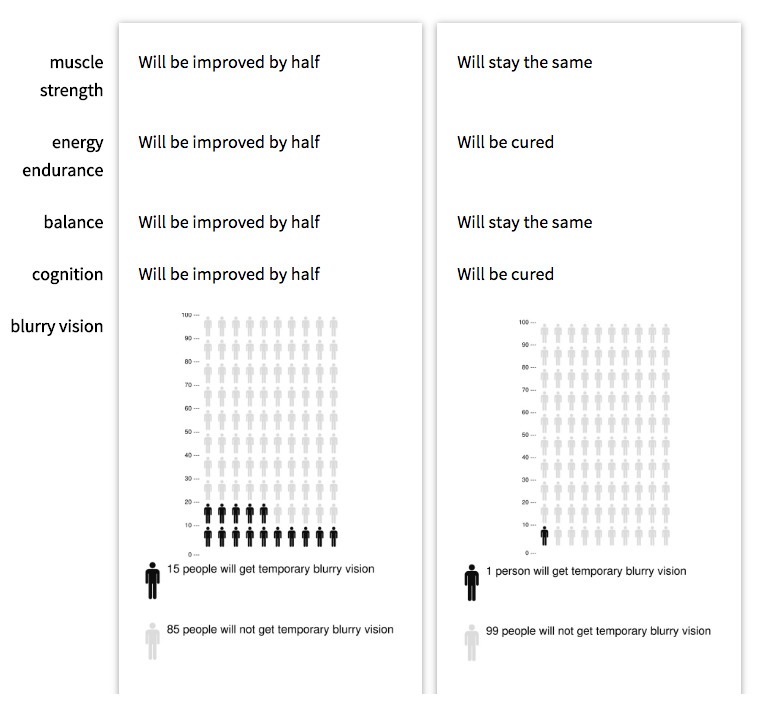
Discrete Choice Experiment – choice task example.

In a DCE respondents are offered a series of choice tasks where they are asked to choose between two or more alternatives in each choice task. In DCEs it is assumed that alternatives can be described by attributes and respondent’s preferences depend upon the levels of the attributes (e.g. one oral tablet a day or 10% risk of mild side effects). This method can for example provide insights about the relative importance of attributes and the trade-offs respondents make between these attributes. DCEs have become one of the most popular methods for eliciting health preferences ^30– 32^. Figure 5 depicts a DCE example choice task where respondents can select their preferred alternative from the two presented alternatives. 

***E-Learning.*** To facilitate the participants understanding of the different type of questionnaires to be completed and what to expect from the whole study itself, a set of e-learning modules will be included. These e-learning modules are based on two components: 1) Introduction to explain why patients are participating; and, 2) Instruction manuals (one for each specific elicitation method). This educational tool will be provided as fluent storylines of about 5 minutes long each with voice-over in English reading aloud the provided script. This script was developed and pre-tested with patient representatives supporting the research team.

***Comprehension assessment scale.*** After completing each preference elicitation method, participants will be asked to score the level of understanding and agreement of the questionnaire using a Likert-type scale ( Table 1). Similar questionnaires have proven to be informative when analyzing study feasibility and heterogeneity of preferences and will provide additional information to answer the methodological research question to compare the three different preference methods ^33, 34^.

**Table 1.** Comprehension assessment scale.

What did you think about the length of this last exercise? -Too Long -Long -Manageable -Just Right
How easy or difficult was it for you to understand the questions? -Very Easy -Easy -Not easy or difficult -Difficult -Very Difficult
How easy or difficult was it for you to answer the questions of this last exercise? -Very Easy -Easy -Not easy or difficult -Difficult -Very Difficult
Did you find the initial video with the cough medicine example useful? -Not at all -A little -Moderately -Very much -Not sure

### Data analysis

***Sample size.*** At the time of this submission, a total of 1050 responses are expected and distributed in the following manner: 300 participants in Group 1, 400 participants in Group 2 and 350 participants in Group 3. This will allow an almost even distribution of the sample between each group and enough numbers completing each type of treatment preference method. From the three methods chosen, the method requiring the largest sample size is the Discrete Choice Experiment (DCE). Calculating the minimal required sample size for a DCE requires prior information about the parameters. As no pilot study has been completed yet, we will start using a rule of thumb [sample size > 500 l / TA], where the sample size of a DCE study depends on the number of choice tasks (T), the number of alternatives in a choice set (A), and largest number of levels in any attribute (l) ^35, 36^. As a result, a sample of 125 participants per group should be sufficient to answer our primary clinical objective. Taking our secondary objectives into account and following DCE practice ^36, 37^ we aim to recruit a sample size of 300 participants completing a DCE exercise ^35^. Once we have data available from our pilot study, using the size calculation approach of De Bekker-Grob *et al*. ^36^ we will be able to confirm whether our sample size of at least 300 respondents is large enough to be able to find differences between attribute levels with a significance level of 0.05 and a power of 0.8 and if the experimental design seems sufficient to elicit trade-offs.

***Statistical analysis plan.*** Descriptive data will be used to report participants’ characteristics. Appropriate tests of differences (Fisher’s exact test, chi-squared or t-test) will be used to establish whether there are any significant differences between sub-groups.

Participants will have to choose an option in order to finish and close the questionnaire. However, in case of missing responses, it will be treated as missing data and further sensitivity analysis will be performed.

***Q-methodology.*** After the Q-methodology (individuals’ unique viewpoints captured in how they ranked the statements) are collected from a sample, they are then statistically correlated in order to reveal clusters of similar viewpoints. The Q-methodology are subjected to a factor analysis, in order to reveal the number of ways the statements were sorted and to identify clusters of respondents whose Q-methodology are highly correlated. Common factor extraction methods include centroid component extraction (i.e. centroid factor analysis) and principle component extractions. Common factor rotation methods include manual rotation and varimax rotation. Factor rotation ensures that each factor offers the best, or most meaningful, vantage point through which the Q-methodologies position and viewpoint can be observed to be closely approximating a particular factor.

The number of Q-methodology associated with each factor will be identified, with a minimum of two Q-sorts required to identify a factor as a shared viewpoint. Essentially, participants with similar views will correlate with the same factor. The extent to which each Q-sort exemplify, or are typical of, each factor is called the ‘”factor loadings” and it explains what percentage of the variance in a particular Q-sort is being accounted for by a particular factor. The number of factors in the final set depends on the variability of the entire sample. Additionally, a composite sort is computed based on weighted averages that represents how a hypothetical respondent that agreed completely with that particular factor (said to have a 100% “loading” on that factor) would have ordered all the statements. This is called a “factor estimate” and it creates a preference “profile”, representing one particular viewpoint that many participants have affiliated with, and aids in the interpretation of why participants might formulate their particular preferences. The comments of respondents, which are strongly associated with a particular factor, can be analysed for motivation and explanation.

A correlation analysis can also be conducted in order to discover whether the results of pooled data across countries are also evident in individual countries.

***BWS2.*** Model-based analysis methods, assuming that the researcher is more interested in the attribute levels and not necessarily best-worst pairs as the main outcome, including, for example, weighted least squares (WLS), multinomial logit model (MNL), mixed logit model (MXL) and (scale-adjusted) latent class model (SALC or LCM) ^38^. In our study, WLS will be used as relatively simple regression model to explore the best-worst data. With WLS, which can be considered as an extension of the well-known ordinary least squares (OLS) regression method, a standard regression model is estimated while using the choice frequencies of the attribute levels as weights in the WLS regression. MNL will be used as an analysis method because it is frequently used in choice modelling to gain insights into the importance of attribute levels without taking scale and preference heterogeneity into account. More sophisticated models like for example MXL and/or LCM will also be used to account for observed preference heterogeneity. A scale-adjusted latent class model will also be estimated to account for observed heterogeneity in scale. These models will be estimated using Pythoniogeme, R and STATA. Estimates from these models will be used to calculate the relative importance of attribute levels, both for subgroups or the entire sample.

***DCE.*** Several models exist to analyse discrete choice data ^39^. Given our interest in accounting for systematic preference heterogeneity (i.e., to determine whether NMD treatment preferences depend on specific respondent characteristics), while also taking scale effects and our sample size into account, led to the decision to employ a multinomial logit model (MNL) with error term heteroscedasticity (or scale variation in which the variability of a variable is unequal across the range of values of a second variable that predicts it) and observed preference heterogeneity or to employ a scaled Latent Class Model (LCM) to analyse the choice observations. Using BPythoniogeme or NLogit Software and taking the best model fit into account based on the Bayesian Information Criterium (BIC), the observations will be analysed. We will use a three-step approach to determine the optimal utility function. First, we will test a number of different specifications for the utility function (i.e. categorical or numerical attribute levels, two-way interactions between attributes, several attribute transformations). Second, we will add and test a number of different scale components to the utility function. Finally, we will allow for several covariates (respondent characteristics) to enter as interactions into the utility function.

For the coefficients, the statistical significance (p-value ≤0.05) indicates that respondents consider the attribute important in making their choices in the DCE. The sign of the coefficient reflects whether the attribute has a positive or negative effect on utility. In terms of the scale parameters, statistically significant parameter estimates indicate the degree to which the associated covariate captures more (positive parameter) or less (negative parameter) consistency in choices.

We will calculate the minimum acceptable benefit (MAB) and maximum acceptable risk (MAR) based on the estimated DCE coefficients. A MAB value represents how much one is willing to benefit for a one-unit change in a risk attribute, and is calculated by taking the ratio of the parameter for a risk attribute to the parameter related to a benefit attribute (or vice versa in case of MAR). For the LCM however, several potential MAB and MAR measures can be derived. Firstly, it is possible to calculate the conditional class MAB (or MAR) values. In the model, each class will have a set of parameter estimates. Whilst of theoretical interest, such MAB and MAR measures are likely to be of limited relevance to the analyst. This is because the LCM assumes that respondents belong to all classes up to a probability (and not to just one class). It is possible however to obtain overall MAB and MAR measures by weighting the conditional MAB or MAR values by the probability that respondents belongs to a given class (given by the class assignment probability outcomes of the LCM). In this study, we will use the latter, also called marginal MAB or MAR values. We will also compute the confidence intervals based on the individual specific MAR and MAB estimates using the Krinsky and Robb procedure, Delta method and/or Bootstrapping.

More detailed and additional information about the analysis will be outlined in a separate statistical analysis plan (SAP), which will be finalized prior to data analysis. This will include additional information about the data cleaning, consistency/validity checks, modeling strategy, and modeling diagnostics.

## Conclusions

The field of PPI is evolving and significantly relevant to inform pharmaceutical research and the drug development process. Understanding the current needs and expectations of patients is particularly necessary in the case of rare diseases and therefore it is important to understand what patients view as important and what level of risk(s) they are willing to tolerate in exchange of certain benefits. This study aims to collect this information while also assessing particular methodological strategies that could facilitate PPI assessments in groups that characterize as rare (i.e. proportionally small samples) and that may present cognitive limitations. The design of this study has followed a patient-centric approach having a group of four patient representatives informing and supporting the study since early stages, and partnering with strong patient organizations. Overall, the findings of this study will inform the NMD community and other stakeholders along the medical product life cycle about treatment preferences as defined by patients themselves.

### Study limitations

Due to the nature of these diseases, there is a high risk of coming short on sample recruitment numbers, however we expect that with the international approach planned and the collaboration of all patient organizations and patient registries supporting this study the risk has been reduced. In case of significantly lower participants, there is a possibility to extend the study and do a second call of recruitment within all those that have not participated before.

The online design of the study will naturally exclude participants with limitations to access a computer or an Internet platform. Alternative options to deliver the survey (e.g. paper copies) could have been considered. However we decided not to allow these to prevent a cofounding factor in responses and to support the delivery of the study internationally within a defined time frame.

The treatment preference methods selected for this study differ in style and the type of PPI that can be obtained from each method. This will limit the amount of parameters that can be compared between each other, however outcomes such as: variance in results, completion rates, participants’ perceptions of the methods and their sensitivity to detect heterogeneity in responses, among others, will allow comparison between methods and results to be informative for the PREFER Project aims.

## Reporting of results

Abstracts, presentations and manuscripts will be prepared in accordance with the PREFER guidelines for publication and dissemination. PREFER consortium members will have the right to review at least 30 days prior to any publication or presentation.

Results of this study will be submitted for publication in peer-reviewed journals in accordance with recommendations of the International Committee of Medical Journal Editors (ICMJE). Depending on the nature of the manuscript, these submissions will be overseen by either: the study Principal Investigator or PREFER work package leaders. Authorship will be determined by mutual agreement and in compliance with the PREFER publication policy. Authors will ensure that the Innovative Medicines Initiative (IMI) and PREFER project is acknowledged and disclaimers are used where necessary.

The PREFER project and study Sponsors support open access to peer-reviewed publications. The green open-access policy or higher will be ensured by parallel publishing in appropriate university library database(s). A summary of the study results will be written for lay audiences and made available to study participants and relevant patient organisations for distribution on their own channels as they decide to (e.g. website, social network platforms, newsletters, patient days, etc). Findings of the study will also be presented at patient conferences and networking events.

Data collected and coded from this study will be of open access for all partners associated with the PREFER Project to facilitate study results dissemination through different channels.

## Patient engagement and involvement

Patient engagement is an integral element of this project. Since the early stages of study design and planning, four patient representatives (two caregivers and two affected patients) have been part of the team and consulted on every aspect of protocol design and development. Additionally, three of the research team members’ have been working closely with the patient organisations and are involved in patient engagement tasks on a daily basis (e.g., support on the co-design of this study, participation in focus groups and advice on dissemination strategies). The PREFER consortium also has an assigned panel leading the development of patient communication material and SOPs.

## Data availability

### Underlying data

No underlying data are associated with this article.

### Extended data

Newcastle University: Supplementary materials for a protocol for quantifying patient preferences in neuromuscular disorders. https://doi.org/10.25405/data.ncl.12841112.v1 ^21^.

This project contains the following extended data:

- _Attributes and Levels List (1).docx
- E-advert invitation 05.05.20.pdf. (Invitation to participate in the study.)
- Participant Information Sheet V2.0 (18.02.2020).docx.
- PREFER Infomed Consent (Version 2.0 (2020-02-18).pdf
- PREFER - NMD TEAM.docx. (Members of the NMD team.)

Extended data are available under the terms of the Creative Commons Attribution 4.0 International license (CC-BY 4.0).
