# Supplementary material for: A study protocol for quantifying patient preferences in neuromuscular disorders: a case study of the IMI PREFER Project
Source: Wellcome Open Res. 2020 Oct 23;5:253. [Version 1] doi: 10.12688/wellcomeopenres.16116.1 (PMC8356266; doi:10.12688/wellcomeopenres.16116.1)
Supplement: Peer review history

# Peer review: Reviewer response for version 1

**DOI:** 10.21956/wellcomeopenres.17689.r44940

**Published:** 2021-08-23

**Authors:** Aisling S Carr

**Affiliations:** 1 MRC Centre for Neuromuscular Diseases, UCL Queen Square Institute of Neurology, National Hospital for Neurology and Neurosurgery, London, UK

This paper describes in detail the approach to collecting qualitative information on patient and care-giver preferences in rare neuromuscular disorders. This work will provide novel and valuable insights into the elements of most relevant to the individuals with the studied conditions (with potential for extrapolation to broader neuromuscular disease spectrum) and guide future study design and drug development.  

 Subjective data such as patient preference is a very challenging metric to try and measure. The initial qualitative work and now this iterative extension to measure and evaluate this information in a larger patient cohort is an ambitious project. I agree with other reviewers that recruitment of these numbers will be challenging. But with a highlight motivated research team and a proactive recruitment strategy across all sites, I do not think this is completely unrealistic. 

 I also wonder if delivery of the questionnaire in the outpatient waiting area may be supported by the study design but also potentially facilitate corroborative clinical data collection in a proportion of contributors (allowing for a degree of validation/ correlation with objective assessments). 

 I have no concerns regarding the methodology of this study and am very interested in the output. 

Is the study design appropriate for the research question?

Yes

Is the rationale for, and objectives of, the study clearly described?

Yes

Are sufficient details of the methods provided to allow replication by others?

Yes

Are the datasets clearly presented in a useable and accessible format?

Yes

Reviewer Expertise:

Neuromuscular diseasesNeuroepidemiology

I confirm that I have read this submission and believe that I have an appropriate level of expertise to confirm that it is of an acceptable scientific standard.

# Peer review: Reviewer response for version 1

**DOI:** 10.21956/wellcomeopenres.17689.r44933

**Published:** 2021-08-09

**Authors:** Dorothée Lulé

**Affiliations:** 1 Department of Neurology, Ulm University, Ulm, Germany

This is a quantitative patient preferences study designed as an online survey, with a cross-over design in two different diseases from the neuromuscular disorders disease group, myotonic dystrophy type 1 (DM1) and mitochondrial myopathies (MM). Three different preference elicitation methods will be compared among different participant subgroups (i.e. disease type, disease phenotypes, patients and caregivers), stratified according to patient age at onset < and >= 20 years) at two different time points (T1 and T2) with a 2-week period in between. The study will compare feasibility, level of understanding and validity of results between the three different preference elicitation methods.

 Low sample size might be a major challenge of the study. No pilot study has been completed yet, but a total of 1050 online responses are expected. However, it’s a very elaborate design and participation will require some time, an online access and comprehensive understanding which will substantially limit the target group.

 Some further challenges are the selection of the measures and the definition of the caregivers. Regarding the measures, patient reported outcomes will be used only, e.g. as surrogate of disease severity and as the numeracy measure so no objective marker or expert opinion is provided which may limit the validity of findings. For caregivers, they are asked to contribute based on their judgments regarding the needs of the patient they care for and not about their own personal preferences. However, caregivers can be carriers of the genetic mutation and thus potentially diseased too which will limit their ability to purely judge on the patient’s perspective.

Is the study design appropriate for the research question?

Yes

Is the rationale for, and objectives of, the study clearly described?

Yes

Are sufficient details of the methods provided to allow replication by others?

Yes

Are the datasets clearly presented in a useable and accessible format?

Yes

Reviewer Expertise:

Neuropsychology in the field of neurodegenerative disorders

I confirm that I have read this submission and believe that I have an appropriate level of expertise to confirm that it is of an acceptable scientific standard.

# Peer review: Reviewer response for version 1

**DOI:** 10.21956/wellcomeopenres.17689.r42352

**Published:** 2021-03-25

**Authors:** Nicholas E Johnson

**Affiliations:** 1 Department of Neurology, Virginia Commonwealth University Health, Richmond, Virginia, USA

This study is designed to assess patient preferences in two different disorders, myotonic dystrophy and mitochondrial disease, with related features despite distinct pathophysiology. The study will use a qualitative approach to solicit these preferences, specifically around risk benefit assessments, to inform drug development. The study is well designed and should it complete will meet the stated objectives. It has the potential to be impactful to the community.  The major limitation is sample size, and the risk of falling short in an ambitious study. It would be ideal to specify additional recruitment approaches.

Is the study design appropriate for the research question?

Yes

Is the rationale for, and objectives of, the study clearly described?

Yes

Are sufficient details of the methods provided to allow replication by others?

Yes

Are the datasets clearly presented in a useable and accessible format?

Yes

Reviewer Expertise:

Muscular dystrophy.

I confirm that I have read this submission and believe that I have an appropriate level of expertise to confirm that it is of an acceptable scientific standard.
